# iPSC-Derived Endothelial Cells as Experimental Models for Predictive and Personalized Strategies in Cardiovascular and Cerebrovascular Disease

**DOI:** 10.3390/ijms27020780

**Published:** 2026-01-13

**Authors:** Lorenzo Fontanelli, Alessio Castronovo, Carolina Ferri, Federico Vozzi, Fabio A. Recchia, Andrea Borghini

**Affiliations:** 1 Health Science Interdisciplinary Centre, Sant’Anna School of Advanced Studies, 56124 Pisa, Italy; alessio.castronovo@santannapisa.it (A.C.);; 2 Department of Biology, University of Pisa, 56126 Pisa, Italy; c.ferri11@studenti.unipi.it; 3 CNR Institute of Clinical Physiology, 56124 Pisa, Italy; federico.vozzi@cnr.it; 4 Section of Cardiology, Department of Medicine, University of Chicago, Chicago, IL 60637, USA

**Keywords:** endothelial cells, induced pluripotent stem cells, personalized medicine, cardiovascular diseases, cerebrovascular disease

## Abstract

Endothelial cells (ECs) regulate vascular homeostasis, and their dysfunction is a key driver of many cardiovascular and cerebrovascular diseases. Human-induced pluripotent stem cell-derived endothelial cells (hiPSC-ECs) provide access to patient-specific vascular cells that can be directed toward arterial, venous, or organotypic phenotypes, enabling personalized in vitro modeling of endothelial pathology. In this review, we discuss how patient-specific iPSC-ECs are used as predictive and personalized two- and three-dimensional models to dissect disease mechanisms and prioritize targeted therapies. We highlight some limitations of this methodology and outline future directions for integrating iPSC-EC-based assays into individualized treatment algorithms.

## 1. Introduction

Endothelial cells (ECs) form a thin, single-cell layer, known as the endothelium, that lines the interior surface of all blood and lymphatic vessels, as well as the heart chambers [1,2]. These specialized cells control the vessel tone, mediate the exchange of oxygen, nutrients and metabolic waste products, and form a barrier between bloodstream and vascular or pericapillary tissues, thus preventing thrombosis [3].

ECs are not a uniform population; instead, their phenotype aligns with their specific functions: arterial ECs are long and narrow, oriented along the direction of blood flow to withstand high shear stress, while venous ECs are wider and flatter with less regularly organized tight junctions [4]. In the brain, ECs form a continuous, non-fenestrated monolayer with complex tight junctions and minimal transcytosis, constituting the structural basis of the blood–brain barrier [5]. By contrast, endocardial ECs are larger and possess numerous microvilli projecting into the cardiac cavity, which significantly increase the luminal surface area, suggesting a sensor role for the endocardium in detecting changes in the cardiac environment [6]. It has been extensively described that maladaptive endothelial responses contribute to a broad spectrum of pathologies, including atherosclerosis [7], microvascular complications of diabetes [8], pulmonary hypertension [9], stroke [10], and neurodegeneration [11]. Endothelial dysfunction is central to both cardiovascular and neurovascular disease: hallmark features include impaired nitric oxide signaling [12], elevated reactive oxygen species [13], altered expression of adhesion molecules, and abnormal permeability [14]. Advances in stem cell biology now allow the generation of endothelial cells from human induced pluripotent stem cells (hiPSC-ECs), which can be further directed towards arterial, venous or organotypic phenotypes using defined patterning cues and microenvironmental conditioning. These patient-specific hiPSC-ECs provide a powerful platform to dissect endothelial dysfunction, model inherited and acquired vascular diseases, and evaluate drug responses, thereby supporting predictive and personalized medicine. An important strength of using differentiated cells derived from human iPSCs is that they retain the complete patient-specific genotype, allowing disease-relevant phenotypes and drug responses to be studied in a way that is difficult to achieve with animal models or heterologous cell lines [15]. However, reprogramming to pluripotency is accompanied by extensive remodeling of DNA methylation and chromatin, largely resetting epigenetic age and erasing many somatic, age- and environment-associated epigenetic signatures, which can limit the ability of iPSC-derived cells to fully capture late-onset or exposure-driven disease mechanisms [16].

iPSC-ECs may anticipate how the endothelium of the patient reacts to an insult. Since iPSC-derived endothelial cells retain the donor’s genetic background and current multi-omics approaches combined with advanced computational tools enable pathway-level analyses, it is now feasible to investigate patient-specific factors underlying endothelial dysfunction and therapeutic responses in controlled experimental settings.

In this review, we will focus on how hiPSC-derived ECs can be used to recapitulate maladaptive responses in vitro, and how such models can support precision medicine strategies and individualized therapy selection. We will highlight technical and conceptual limitations, and describe how hiPSC-EC models may be integrated into future workflows for individualized risk stratification and therapy selection.

## 2. Endothelial Cells from iPSCs: Differentiation and Characterization

In the last decade, several protocols have been developed to generate ECs from iPSCs, recapitulating embryonic vasculogenesis via the mesodermal progenitor stage [17]. During embryonic development, ECs derive from mesodermal precursors, which establish a primitive vascular plexus that, during vasculogenesis, is remodeled into a mature hierarchy of arteries, veins, and capillaries [18]. Various developmental signaling pathways lead ECs to specialize in their specific functions. Arterial specification is driven by VEGF–Notch signaling: high VEGF levels activate VEGFR2, which, by inducing the Notch pathway, promotes arterial differentiation and suppresses venous traits [19]. Conversely, venous identity is promoted by transcription factors, such as NR2F2, which represses Notch signaling and supports the venous gene program [20]. Hemodynamic forces further contribute to the differentiation as arterial and venous ECs: laminar shear stress promotes arterial differentiation of hiPSC-ECs by upregulating arterial genes such as EFNB2 and downregulating venous markers via mechanotransduction pathways that augment Notch signaling [21]. Blood–brain barrier endothelium, on the other hand, is induced and maintained by cues from the neural microenvironment: in particular, Wnt/β-catenin signaling from glial and neural cells induces CNS endothelium to acquire barrier properties, upregulating tight-junction genes such as *CLDN5* during brain vascular development [22].

ECs are characteristically recognized by the expression of specific markers, including VE-cadherin, platelet endothelial cell adhesion molecule-1 (PECAM-1/CD31), von Willebrand factor (vWF), vascular endothelial growth factor receptor 2 (VEGFR2), and adhesion molecules such as ICAM-1 and VCAM-1, which collectively reflect their identity, barrier function, and activation state in the vascular system [23]. In vitro, ECs can be generated from hiPSCs within 4 to 15 days, depending on the protocol, with typical cytokine-induced differentiation requiring about 8–10 days; faster methods employ transcription factor ETV2, shortening the differentiation process to 4 days, followed by expansion for subsequent use [24,25]. Endothelial arterial phenotype is primarily induced by VEGF and Notch signaling. High VEGF-A levels activate Notch by upregulating the ligand DLL4 and induce arterial markers such as ephrin-B2, NRP1 and HEY1/2, while lower VEGF permits the venous program driven by NR2F2 [26]. Indeed, Notch signaling is crucial for arterial specification, as Notch inhibition diverts differentiating cells to a venous fate, even in the presence of VEGF [26]. cAMP agonists synergize with VEGF to enhance Notch activity and concomitantly suppress the venous regulator NR2F2, thereby accelerating the arterial phenotype differentiation [21]. Shear stress-sensitive ion channels play a central role in how ECs sense blood flow and regulate vascular tone. In native endothelium, mechanosensitive channels transduce fluid shear into changes in membrane potential and intracellular calcium, which activate endothelial nitric oxide synthase (eNOS), promote nitric oxide release, and thereby drive flow-mediated vasodilation [27,28]. iPSC-derived ECs recapitulate key aspects of this mechanotransduction cascade, as exposure to physiological shear stress induces endothelial alignment, enhances nitric oxide production, and upregulates arterial markers, resulting in the acquisition of more mature, flow-responsive biophysical and functional properties [29].

Several protocols have been established to generate iPSC-ECs, but it must be noted that certain genetic backgrounds may influence differentiation. In their study, Carcamo-Orive and colleagues [30] found that variations in the expression of homeobox transcription factors HOXA5 and HOXC10, which depends on donor genetic background, influence developmental programs that govern mesodermal and vascular lineage commitment and thus differentiation efficiency toward the endothelial lineage, potentially affecting patient-specific disease modeling outcomes. Also epigenetic factors influence the differentiation of iPSCs: during the generation, iPSCs undergo extensive reprogramming that erases most of the epigenetic signatures of the subject. However, some residual “epigenetic memory” from the donor cell can persist, particularly in early-passage iPSC lines. This residual memory can influence their differentiation potential, favoring lineages related to the donor tissue. Such epigenetic memory is typically lost along cell passaging, as iPSCs progressively acquire a more fully reprogrammed, higher state of pluripotency [31].

Furthermore, stochastic effects may impair reproducibility of the findings: in the same study, Carcamo-Orive and colleagues [30] describe how the Polycomb Repressive Complex (PRC), an epigenetic regulator that represses gene expression via trimethylation of histone H3 lysine 27 (H3K27me3), may influence cell differentiation. In particular, PRC target genes exhibited increased non-genetic transcriptional variability across iPSC lines, reflecting heterogeneity in epigenetic silencing states during reprogramming and maintenance of pluripotency. Such epigenetic variability can influence differentiation trajectories and cellular phenotypes by controlling access to developmental genes, thereby introducing non-genetic sources of functional variability in iPSC-based disease models. It should be noted that Manganelli et al. demonstrated, for the first time, that reprogramming somatic cells into iPSCs leads to the constitutive expression of the progesterone receptor. Considering the crucial roles of progesterone in vasodilation, angiogenesis, vascular permeability, neuroprotection, and blood–brain barrier stabilization, it is important to understand its role during iPSC differentiation [32].

## 3. Patient-Specific iPSC-ECs as Predictive and Personalized Models for Cardiovascular Applications

The idea that ECs could be used as patient-tailored experimental models was elegantly illustrated by Jiménez-Torres and colleagues [33]. In their seminal work, the authors demonstrated that it is possible to isolate patient-specific ECs from renal cell carcinoma (RCC) samples to model and test anti-angiogenic treatments in vitro. Tumor-associated endothelial cells (TEnCs) and normal endothelial cells (NEnCs) from each patient’s tumor and adjacent non-tumor kidney tissue, sorted by CD31-based magnetic assay, were used to construct organotypic microvessels mimicking in vivo blood vessels. The authors demonstrated that TEnC vessels exhibited higher angiogenic potential than their normal counterparts, forming significantly more sprouts and showing disorganized endothelial layers with reduced VE-cadherin expression, reflecting the pathological phenotype of tumor vasculature. Quantitative PCR and ELISA revealed that TEnC vessels expressed and secreted higher levels of pro-angiogenic molecules, including VEGF-A, VEGF-C, and FGF-2, and increased the matrix metalloproteinases (MMP) MMP-2 and MMP-14, enzymes involved in extracellular matrix remodeling and invasion. Conversely, adhesion-related genes such as *CDH5* (VE-cadherin) were downregulated. Importantly, when these personalized microvessels were exposed to the anti-angiogenic drugs sunitinib and pazopanib, heterogeneous responses were observed among patients and between TEnCs and NEnCs. In some patients, TEnC vessels responded to sunitinib with decreased sprouting, while others were resistant or even showed increased sprouting. These variable outcomes paralleled the clinical heterogeneity in drug response, demonstrating the model’s predictive potential for personalized therapy selection.

More recently, iPSC-ECs generated from patients with familial hypercholesterolemia (FH) due to LDLR mutations were found to display reduced LDL uptake, impaired angiogenic capacity, and heightened inflammatory and oxidative stress signatures, providing a platform to interrogate endothelial susceptibility to atherosclerosis and to test lipid-lowering or anti-inflammatory interventions in a patient-specific manner. In their study, Zakharova and colleagues [34] generated iPSCs from healthy donors and from two heterozygous FH patients carrying clinically characterized pathogenic LDLR variants affecting receptor trafficking and recycling. Then they directed these lines through a three-step mesoderm–endothelium differentiation protocol followed by CD31-based magnetic enrichment to obtain highly pure ECs (VE-cadherin^+^, CD31^+^, vWF^+^). At the protein level, normal iPSC-ECs predominantly expressed the mature LDL receptor, whereas FH iPSC-ECs showed a marked reduction in the mature form, with one line accumulating immature LDLR in the endoplasmic reticulum and the other displaying an overall depletion of both immature and mature receptor species; functionally, this translated into almost complete loss of fluorescent LDL uptake in FH-derived endothelium despite preserved endothelial identity. This almost complete loss of LDL uptake in FH-derived iPSC-ECs, despite preserved endothelial identity, closely mirrors the fundamental cellular lesion in familial hypercholesterolemia, in which loss-of-function mutations in LDLR abolish high-affinity LDL binding and internalization [35,36]. RNA-seq comparison between control and FH-derived iPSC-ECs identified 39 differentially expressed genes, with downregulation of monocarboxylic acid transporters and genes involved in exocytosis, cell–cell adhesion and responses to external stimuli, and upregulation of genes related to cell secretion and leukocyte activation, including dysregulation of angiogenesis- and Rap1 pathway-associated genes. Interestingly, Rap1 maintains endothelial barrier function, angiogenesis, and vascular homeostasis [37] and protects against atherosclerosis in murine models by limiting inflammatory NF-κB-driven signaling [38]. Taken together, these results indicate that endothelial cells with *LDLR* mutations are intrinsically predisposed to dysfunction and chronic inflammation, regardless of systemic cholesterol levels, thereby providing a mechanistically informative iPSC-EC platform for studying FH-associated atherogenesis and screening therapies aimed to correct *LDLR* processing or mitigate endothelial injury.

EC-based models have also been employed to investigate acquired diseases, thereby enabling assessment of the contribution of the patient’s genotype. Zhou and colleagues investigated the use of iPSC-ECs derived from patients with anti-complement factor H autoantibody-associated atypical hemolytic uremic syndrome (aHUS) [39], an acquired autoimmune disease, with a high genetic predisposition, caused by dysregulation of the complement system [40]. In patients with anti-complement factor H autoantibody-associated atypical hemolytic uremic syndrome (aHUS), these autoantibodies impair complement factor H normal regulatory function in the blood, leading to excessive complement activation on the endothelial surface. The outcome is an increased deposition of complement components, such as C3b, and the formation of the membrane attack complex, which induces endothelial cell injury, apoptosis, and subsequent thrombotic microangiopathy. Notably, the authors demonstrated that patient-derived ECs exhibit intrinsic endothelial defects, characterized by impaired migration, tube formation, and proliferation, as well as aberrant activation of key signaling pathways compared with control-derived ECs. In detail, impaired p38 MAPK signaling emerged as a pivotal molecular hallmark of disease. p38 MAPK is known to maintain endothelial integrity partly by regulating the expression of VE-cadherin [41], which is essential for cell–cell adhesion and vascular homeostasis [42] and was also found to be downregulated in aHUS iPSC-ECs. This platform was then used to test the effect of anisomycin, a specific p38 MAPK activator [39]. Anisomycin effectively restored p38 phosphorylation levels and rescued endothelial dysfunction phenotypes, improving cell migration and tube formation in the patient-derived iPSC-ECs. Additionally, overexpression of *MKK6*, a kinase that activates p38 signaling, also significantly enhanced p38 activity and endothelial function. At the transcriptomic levels, RNA-seq identified 1401 differentially expressed genes in aHUS-derived ECs, including downregulation of *PECAM1*, *BMP4*, *HOXA3*, and *CDH* genes involved in blood vessel development, cell migration, and angiogenesis [39,43,44,45]. Moreover, while both ECs derived from patient-specific iPSCs and those derived from controls were vulnerable when exposed to serum from aHUS patients containing anti-complement factor H autoantibodies, the former showed a more pronounced aberrant response to complement dysregulation.

The damage caused by aHUS diseased serum was complement-dependent, as blocking complement with compstatin rescued endothelial dysfunction. Thus, the iPSC-EC model effectively recapitulates this pathological process, showing that patient serum triggers complement-mediated cytotoxicity, characterized by disruption of endothelial barrier function, increased apoptosis rates, and exacerbated inflammatory signaling. These molecular and cellular events mimic the in vivo endothelial damage observed in aHUS, validating the use of iPSC-ECs to mimic disease-relevant endothelial pathobiology.

Further studying acquired diseases, Gorashi and colleagues developed an in vitro model of diabetic endothelial dysfunction [46]. ECs derived from diabetic patient iPSCs expressed inflammation-related markers such as VCAM-1, ICAM-1, and P-selectin, showed higher intracellular ROS levels, and had a higher proportion of platelet-adherent cells compared with ECs derived from control iPSCs. At the transcriptomic level, 3464 genes were differentially expressed, including those involved in angiogenesis and vascular development. Interestingly, when exposed to a medium rich in glucose, urea, and TNF-α, which replicates diabetic plasma, control ECs increased expression of ICAM-1, VCAM-1, and P-selectin, whereas such a response was not observed in ECs derived from diabetic patients, suggesting that these cells may better tolerate the altered microenvironment. To test the feasibility of this model as a platform for drug screening, the authors tested several compounds known to improve endothelial function, such as the PPAR-γ agonist pioglitazone, the angiotensin receptor blockers telmisartan and valsartan, the anticoagulant heparin, aspirin, and resveratrol. The authors not only demonstrated that pioglitazone, telmisartan, and valsartan were the most effective drugs for reducing VCAM-1 expression, but also identified the optimal therapeutic regimen and drug concentration for each patient, thereby generating a precision-medicine framework in which treatment strategies can be precisely tailored.

To investigate the impact of obesity on endothelial dysfunction, Gu and colleagues generated iPSC-ECs from both control and diet-induced obesity mice [47]. Functionally, iPSC-ECs from diet-induced obesity mice exhibited significantly reduced migration, proliferation, increased apoptosis under hypoxia, and diminished tube formation capacity, indicative of endothelial dysfunction. Molecular analyses revealed altered gene expression in obese-derived iPSC-ECs, including upregulation of apoptosis, inflammation, oxidative stress, and cellular senescence pathways. Key molecular findings in obese-derived iPSC-ECs included also reduced NO production and impaired eNOS activity, reflected by decreased eNOS phosphorylation at Ser1177, increased phosphorylation at Thr495 (which inhibits eNOS activity), and enhanced eNOS uncoupling. The authors demonstrated that pravastatin restored NO production by enhancing eNOS activity, thereby decreasing oxidative stress and improving NO bioavailability. Pravastatin enhanced cell migration, proliferation, and tube formation, while significantly lowering apoptosis under hypoxic conditions. These results were later confirmed in vivo, where pravastatin administration improved perfusion and vascular regeneration in a murine hindlimb ischemia model transplanted with obese-derived iPSC-ECs, and decreased muscle damage and inflammation. When transplanted into ischemic hindlimbs, these iPSC-ECs led to poorer reperfusion and greater muscle damage and inflammation than control iPSC-ECs. In line with the previous results, co-administration of pravastatin improved vascular integration and functional recovery, confirming the therapeutic potential of statin-mediated eNOS/NO pathway activation in reversing obesity-induced endothelial dysfunction. This study demonstrated that obesity impairs the regenerative potential and function of iPSC-ECs, highlighting important molecular pathways involving oxidative stress, inflammation, and eNOS regulation. Such insights are crucial for advancing personalized regenerative therapies targeting vascular complications of obesity and diabetes [47].

Similarly, Gu and colleagues [48] demonstrated that patient-specific iPSC-ECs can be used to identify potential therapies for pulmonary arterial hypertension through combined phenotypic drug screening and in silico transcriptomic analysis. Using iPSC-ECs from six patients with pulmonary arterial hypertension, they demonstrated that they had reduced survival under stress, impaired angiogenesis, reduced adhesion to extracellular matrices, and decreased migration. These cells displayed altered bone morphogenetic protein (BMP) signaling pathways, including reduced expression of the BMP receptor BMPR2. The authors then screened 4500 compounds for their ability to improve endothelial survival after serum withdrawal, a common in vitro stress condition, identifying tyrphostin AG1296, a selective tyrosine kinase inhibitor of PDGFR, as the lead compound that enhanced survival and angiogenesis and reversed disease-associated molecular signatures. Mechanistically, AG1296 restored and enhanced BMPR2 signaling, a central pathway disrupted in pulmonary arterial hypertension. It increased the abundance of BMPR1A, BMPR1B, and BMPR2 receptors and activated downstream signaling cascades, including pSMAD1/5-ID1, pAKT, and pERK, leading to improved endothelial survival, angiogenesis, and reduced apoptosis. Transcriptomic analyses showed that AG1296 upregulated pro-survival and pro-angiogenic genes such as *APLN*, *VEGFA*, *BIRC3*, and *FST*, while downregulating genes related to inflammation, smooth muscle proliferation, and apoptosis. Notably, RNA-seq and LINCS-based bioinformatic analysis revealed that AG1296 induced a robust anti-pulmonary arterial hypertension gene signature, characterized by upregulation of *CREB3* and *CREB5*, transcriptional co-activators that enhance SMAD1/5-ID1 signaling and endothelial stability. Functionally, AG1296 not only improved endothelial function but also suppressed pulmonary arterial smooth muscle cell (SMC) proliferation, contributing to the regression of neointimal lesions in ex vivo lung organ culture. In a rat model of severe pulmonary artery hypertension, AG1296 reduced right ventricular systolic pressure and reversed vascular occlusions, demonstrating therapeutic efficacy in vivo. Importantly, compared to other TKIs such as imatinib, dasatinib, axitinib, and pazopanib, AG1296 showed superior endothelial protection and BMPR2 activation without inducing endothelial apoptosis or inflammation.

Since iPSCs allow the generation of patient-specific testing models, they can be used to evaluate the effects of environmental factors in a way that is much faster than a classical epidemiological investigation [49], and to study the interplay between genetic background and external noxious agents [50]. Tang and colleagues used H9 human embryonic stem cells differentiated into endothelial cells (H9-ECs) as a model to study cadmium toxicity [51]. Cadmium is an environmental pollutant, and its exposure is associated with an increase in all-cause and cardiovascular mortality [52]. To evaluate its effects in vitro, H9-ECs were exposed to escalating doses of cadmium chloride (CdCl_2_) for 24 h, which reduced EC proliferation and their ability to form capillary-like tube structures on Matrigel in a dose-dependent manner, indicating impaired angiogenic capacity. In the wound healing scratch assay, CdCl_2_ treatment markedly decreased ECs migration, delaying wound closure compared to controls. RNA sequencing identified a set of differentially expressed genes in H9-ECs treated with CdCl_2_ compared to control cells related to angiogenesis, i.e., vascular development pathways, apoptotic processes, mitochondrial dysfunction and MAPK signaling, notably the p38 and ERK branches. These functional deficits in angiogenesis, migration, and proliferation mirror the endothelial dysfunction due to cadmium toxicity, supporting the model relevance to vascular injury. Authors then tested the molecules SB203580 and PD0325901, respectively, targeting the p38 and ERK pathways, demonstrating that inhibition of these pathways mitigated apoptosis and partially restored EC function [51].

iPSC-ECs have also been used to study how inherited and epigenetically encoded factors contribute to vascular aging and inflammatory responses in coronary artery disease. As a multifactorial disease, genetic predisposition and lifestyle significantly influence disease development and progression. The group of Landmesser generated iPSCs from lifestyle-matched donors stratified by cardiovascular risk (older healthy individuals, patients with stable coronary artery disease, and younger patients with acute coronary syndrome) and differentiated them into ECs, which were then stimulated with TNF-α to mimic an inflammatory state under static, atheroprotective, and atheroprone flow conditions. Compared with cells from healthy donors, iPSC-ECs from acute coronary syndrome patients tended to show stronger TNF-α-induced upregulation of adhesion molecules such as E-selectin and ICAM-1, accompanied by increased monocyte recruitment [53] even under laminar, classically protective flow patterns, while VCAM-1 responses were comparatively blunted [54].

iPSC-ECs from higher-risk donors exhibited also more rapid telomere shortening with each population doubling, consistent with features of accelerated endothelial senescence [54]. Transcriptomic profiling further indicated that TNF-α elicited distinct gene-expression programs in cells from acute coronary syndrome versus healthy donors, with differential enrichment of pathways involved in leukocyte and lymphocyte regulation, adaptive immunity, and cytokine signaling. Moreover, epigenetic “mitotic age” estimates derived from DNA methylation also increased following endothelial differentiation in all groups, suggesting that these in vitro models can capture aspects of proliferative history relevant to vascular aging [53]. These pioneering findings demonstrate that iPSCs serve as a viable in vitro model for polygenic diseases such as coronary artery disease [53,54].

**Figure 1:**
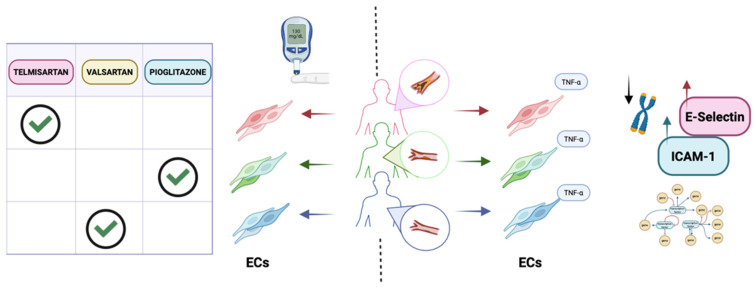
Endothelial cell models of cardiovascular disease. (**Left**): ECs derived from patients with diabetes were used to identify an optimal antidiabetic strategy. Patient-derived ECs can be generated and screened for their response to multiple drugs and to assess treatment responses. Gorashi and colleagues exposed these ECs to different PPAR-γ agonists and measured reduced VCAM-1 expression as a proxy for diabetes-associated vascular dysfunction. (**Right**): ECs can model how cardiovascular risk shapes endothelial inflammatory responses. Landmesser and colleagues generated iPSCs from lifestyle-matched donors stratified by risk (older healthy individuals, stable coronary artery disease, and younger acute coronary syndrome) and differentiated them into ECs, then applied TNF-α under static, atheroprotective, or atheroprone flow. Compared with healthy donor cells, acute coronary syndrome iPSC-ECs showed greater TNF-α-induced upregulation of adhesion molecules (E-selectin, ICAM-1) and distinct transcriptomic signatures. Created in Biorender. Fontanelli L. et al. (2025) https://BioRender.com/ (accessed on 19 December 2025).

Together, these observations support the use of iPSC-ECs as experimentally tractable, patient-specific vascular surrogates that reflect individual cardiovascular risk profiles and offer a reliable model for predictive and personalized testing in cardiovascular disease, accelerating the identification of therapeutic targets and the development of predictive, individualized treatments (Figure 1 and Table 1).

## 4. Patient-Specific iPSC-ECs as Predictive and Personalized Models for Cerebrovascular Studies

Cerebral Autosomal Dominant Arteriopathy with Subcortical Infarcts and Leukoencephalopathy (CADASIL) is a rare hereditary cerebrovascular disorder caused by mutations in the *NOTCH3* gene. It primarily affects the small blood vessels in the brain, leading to thickening of vessel walls and reduced blood flow, which results in recurrent subcortical ischemic strokes, cognitive decline, and progressive dementia [55]. The mutant NOTCH3 protein in vascular smooth muscle cells (VSMCs) and pericytes leads to loss of mural cells and capillary dysfunction.

To study CADASIL in vitro, Kelleher and colleagues [56] generated iPSCs from patients and differentiated them into both ECs and VSMCs. Co-culture experiments with patient-derived cells revealed that CADASIL mutant VSMCs showed an inability to support microvessel network formation. Specifically, CADASIL iPSC-derived VSMCs had lower expression of PDGF receptor-β and secreted less VEGF, impairing the survival and stability of capillary structures. As a result, when patient-derived endothelial cells and VSMCs were combined, the capillary-like networks were unstable and prone to degeneration, reflecting the small-vessel loss observed in patients. Authors then tested tailored interventions on this model, adding back VEGF to the cultures, or using siRNA to knock down mutant *NOTCH3* in patient cells. Both strategies partially restored capillary stability. In detail, supplemental VEGF improved endothelial network formation, and reducing NOTCH3 activity in VSMCs mitigated its toxic gain-of-function effect, allowing for more normal vessel development. These findings highlighted the molecular pathway by which *NOTCH3* mutations cause the disease and suggested potential therapeutic interventions. The iPSC-EC model can help in predicting the beneficial effects of drugs utilized to treat other diseases in specific patients. For example, CADASIL cells can be tested with various Notch pathway modulators or with metabolic treatments to observe their impact on in vitro vessel integrity and guide off-label therapy tailored to every single patient’s molecular defect.

Moyamoya disease (MMD) is a rare cerebrovascular disorder marked by progressive stenosis of the intracranial internal carotid arteries and development of fragile collateral vessels at the base of the brain [57]. In this disease, dysregulation of ECs results in altered vessel repair and maladaptive formation of new blood vessels, contributing to the characteristic “puff of smoke” appearance on angiography. Patient-specific iPSC-derived ECs have emerged as a powerful in vitro model to study MMD in the context of an individual’s genetic background. These models allow researchers to investigate endothelial dysfunction in MMD, uncover molecular pathways unique to patients, and test personalized therapeutic strategies. Early iPSC studies provided the first direct evidence of intrinsic endothelial defects in MMD. Hitomi and colleagues generated iPSC lines from MMD patients (carrying the East Asian founder variant RNF213 R4810K) and healthy controls, subsequently inducing their differentiation into vascular ECs [58]. They found that MMD patient-derived iPSC-ECs formed fewer, shorter tube-like structures than controls, indicative of reduced angiogenic capacity. This angiogenic defect was observed even under pro-angiogenic conditions and was one of the first functional phenotypes recapitulated in an MMD cellular model. These results were later confirmed by Hamauchi and colleagues [59]. In co-culture assays, endothelial tubes formed by MMD iPSC-ECs were significantly shorter and fewer than those formed by control ECs, regardless of the presence of vascular growth factors such as VEGF or bFGF. Interestingly, endothelial proliferation rates were similar between MMD and control iPSC-ECs, suggesting that the disease primarily impairs the ability to sprout new vessels rather than general cell growth. Together, these findings indicate an intrinsic endothelial dysfunction in MMD that leads to poor angiogenic response, consistent with clinical observations of inadequate collateral vessel development in patients. At the molecular level, DNA microarrays showed significant suppression of multiple ECM–receptor interaction genes in MMD iPSC-ECs with integrin (ITG) β3 and ITGB8, which mediate endothelial adhesion and angiogenesis.

Since integrins are crucial for endothelial migration and organization during angiogenesis [60], their downregulation provides one explanation for the poor tube-forming ability of MMD cells. In contrast with the ECM genes, 117 genes were upregulated. Pathway analysis pointed to cell-cycle and mitosis-related genes (e.g., *BUB1* and *PTTG1* encoding securin) being enriched in MMD cells. Proteomic analysis further showed increases in nuclear proteins involved in RNA processing (heterogeneous nuclear ribonucleoproteins and splicing factors) and cell proliferation in MMD ECs, whereas certain structural proteins (e.g., caldesmon and cytokeratin-18) were decreased. These shifts suggest that MMD endothelial cells adopt an altered regulatory state, potentially affecting their responses to stress or growth signals.

Notably, iPSC models have helped pinpoint which vascular cell types are most affected in MMD. Using the same patient iPSC lines, Tokairin and colleagues differentiated not only ECs but also VSMCs, the other major component of blood vessel walls [61]. Under baseline conditions, patient-derived iPSC-VSMCs were almost indistinguishable from healthy controls in morphology, marker expression, and functions such as proliferation, migration, and contractility. At the transcriptomic level, only 6 genes were differentially expressed in MMD VSMCs compared to controls, and no significant functional deficits were observed. In contrast, the iPSC-ECs from the same patients showed about 120 gene expression differences and a clear angiogenic impairment. Such side-by-side comparison helped to demonstrate that, at least in vitro, endothelial cells carry the predominant disease signal in MMD and can help identify potential therapeutic targets in this disease.

Arce and colleagues [62] used iPSCs from patients with familial cerebral cavernous malformation due to heterozygous *KRIT1* mutations to generate ECs and self-assembled vascular organoids. The patient-specific organoids developed abnormally dilated, leaky vascular structures resembling cavernomas, and the iPSC-derived ECs showed alterations in the expression of genes such as *KLF2*, *KLF4*, *CAV1*, *ADAMTS4*, and *vWF,* as well as cell junctional defects, resulting in reduced trans-endothelial electrical resistance. Taken together, these results indicate compromised endothelial barrier integrity and are comparable to what is observed in vivo. Interestingly, when the patient-derived ECs were injected into ex vivo mouse brain explants, they successfully integrated into the existing mouse vasculature.

Unlike control ECs, the mutant ECs from cerebral cavernous malformation patients reshaped and remodeled pre-existing vessels, leading to morphological changes, including increased vessel diameter and reorganization of nearby vascular smooth muscle cells. This process has been shown in murine cavernomas [63] and possibly mediated by the upregulation of genes involved in extracellular matrix reorganization, such as *ADAMTS4*.

**Figure 2:**
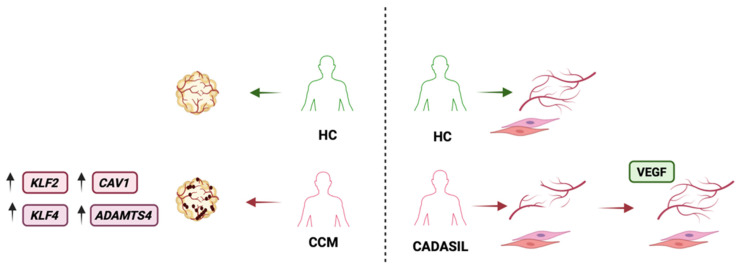
Endothelial cell models of cerebrovascular disease. ECs have been used to model cerebrovascular disorders. (**Left**): Vascularized organoids derived from healthy controls and cerebral cavernous malformation patients. Patient-derived organoids developed abnormally dilated, leaky vascular structures resembling cavernomas and showed dysregulated expression of the associated genes, including *KLF2*, *KLF4*, *CAV1*, and *ADAMTS4*. (**Right**): Kelleher and colleagues differentiated ECs and VSMCs from CADASIL iPSCs and assessed vessel formation in co-culture. CADASIL mutant VSMCs failed to support microvessel network formation, impairing capillary survival and stability; accordingly, patient-derived EC–VSMC networks were unstable and prone to degeneration, consistent with small-vessel loss in CADASIL. Addition of VEGF partially rescued endothelial network formation. CCM: cerebral cavernous malformation; HCs: healthy controls. Created in Biorender. Fontanelli L. et al. (2025) https://BioRender.com/ (accessed on 19 December 2025).

Taken together, these results show that, also in cerebrovascular diseases, iPSC-ECs can serve as patient-specific models, enabling the identification of therapeutic targets and the mechanistic pathways underlying the diseases (Figure 2 and Table 2).

To reduce variability arising from differences in donor genetic backgrounds, CRISPR-Cas9 technology—a highly efficient gene-editing tool—can be employed either to correct mutations in patient-derived cells or to introduce putative causative lesions into iPSCs derived from healthy individuals. This process results in the creation of isogenic cell pairs that differ by only a single genetic modification, allowing for detailed examination of the molecular and cellular phenotypes associated with specific abnormalities. These isogenic cell lines are invaluable not only for understanding the cellular impact of disease mutations, but also for supporting genetic and pharmacological screening efforts to identify underlying pathological mechanisms [64].

## 5. Limitations

Despite the promise of iPSC-ECs as experimental models for personalized medicine, several important technical and conceptual limitations remain. One significant drawback is the erasure of epigenetic memory during the reprogramming process [65], which means that disease-relevant, environmentally acquired epigenetic signatures from the donor are largely lost, reducing the capacity to recapitulate complex, acquired pathological phenotypes, especially those influenced by age or chronic exposures. Additionally, experimental designs often lack precise definition or control of the duration and timing of exposure to pathological stimuli, such as toxins, inflammatory mediators, or disease-causing mutations, making it challenging to model the gradual progression and cumulative nature of many vascular diseases. Furthermore, long-term effects of potential treatments or gene edits remain poorly characterized in vitro due to limitations in culture lifespan, batch variability, and an inherent fetal-like or immature state of many iPSC-ECs. Importantly, the vascular endothelium does not function in isolation; its properties and pathophysiological responses are shaped by interactions with pericytes [66], smooth muscle cells [67], immune cells [68], and neural components [69,70] within the vessel microenvironment as well as the hemodynamic environment reproducing physical forces acting on cells [71,72]. Consequently, truly predictive in vitro models require multicellular or three-dimensional systems that recapitulate cellular crosstalk and tissue context [73]; current monoculture or oversimplified models may miss key mechanistic insights relevant to in vivo physiology and personalized therapeutic strategies. In detail, compared with native endothelial cells, hiPSC-ECs typically exhibit reduced vWF production and predominantly rounded, immature Weibel–Palade bodies, rather than the elongated tubular structures characteristic of fully mature endothelium [24]. Moreover, the limited expansion capacity of these cells poses a challenge for their large-scale production. To overcome this constraint, Aoki et colleagues [74] demonstrated that the use of a defined combination of three small molecules—Y-27632, A83-01, and CHIR-99021, targeting the ROCK, TGF-β, and GSK3β/Wnt pathways, respectively—markedly activates protein synthesis–associated signaling and substantially increases the proliferative potential of hiPSC-derived endothelial progenitor cells, which may represent an expandable intermediate population that can be subsequently driven toward mature, tissue-specific endothelial phenotypes potentially enabling large-scale cell production. Regarding the aforementioned immature state of iPSC-ECs, it must be emphasized that co-culture systems with tissue-specific cell types and three-dimensional models, such as microtissues and organoids, have been shown to promote endothelial specialization by recapitulating key cell–cell interactions and biomechanical cues present in vivo [75].

For example, co-culture with hiPSC-derived cardiomyocytes induces rapid acquisition of cardiac-specific endothelial traits, underscoring the importance of paracrine signaling and direct cellular crosstalk for organotypic endothelial maturation [76]. In this context, physical stimulation strategies are emerging as complementary tools to accelerate endothelial functional maturation. Reviewed studies [77], largely conducted using primary endothelial cells such as human umbilical vein endothelial cells and mesenchymal stem cells, indicate that exposure to electric fields can modulate endothelial migration, proliferation, adhesion, and structural organization. Electrical stimulation has been reported to enhance angiogenic and vasculogenic responses, in part through the regulation of pro-angiogenic signaling pathways, including VEGF, and by promoting the formation and stabilization of capillary-like networks, particularly in three-dimensional culture systems. Although these effects have not yet been directly demonstrated in hiPSC-ECs, the shared endothelial signaling machinery suggests that electric field-based approaches may represent a promising avenue to improve hiPSC-EC maturation and vascular organization, warranting further targeted investigation.

A final element to be considered is the integration of Organ-on-Chip technology: this type of platform can replicate organ-level physiology through a cellular 3D environment, blood flow hemodynamics, mechanical forces, and organ crosstalk. In this context, integrating iPSC-ECs into Organ-on-Chips could support the generation of microphysiological systems that recapitulate specific vascular functions—such as barrier integrity, flow-mediated responses, and multicellular interactions—providing a close-to-realistic setting for studying cardiovascular and cerebrovascular diseases. For cardiovascular diseases, microfluidic platforms model their complex pathophysiology by replicating disturbed hemodynamic conditions and immune inflammation. These systems could incorporate different vessel geometry configurations, endothelial dysfunction, lipid accumulation, and inflammatory cell infiltration [78]. For cerebrovascular ones, the combination of iPSC technology with Organ-on-Chip has enabled the creation of personalized blood–brain barrier chips displaying cellular, molecular, and physiological properties typical of this structure in the in vivo condition [79].

## 6. Future Applications

In the future, with an increased knowledge of cell biology characteristics and a larger availability of patient-derived iPSCs also for the generation of ECs, it is plausible that personalized in vitro models will become increasingly widespread. These models will become more complex, introducing a greater number of interconnected cellular components. For instance, assembloid, i.e., 3D models composed of multiple cell lines, are proving better-suited platforms for drug testing in neurology and cardiology [80]. Patient-derived endothelial models could evolve into clinical decision-support assays for precision cardiovascular and cerebrovascular medicine. One near-term direction is endothelial pharmacotyping, in which patient-specific (or genotype-stratified) iPSC-ECs are challenged with disease-relevant stressors (e.g., inflammatory cytokines, shear perturbation, hypoxia–reoxygenation, or patient plasma) and profiled across a panel of candidate therapies to generate a ranked phenotypic rescue signature, thereby supporting selection of the most suitable medicinal product for the individual. A complementary direction is endothelial risk profiling, where standardized “stress tests” quantify endothelial reserve and vulnerability to dysfunction, potentially informing disease likelihood, penetrance, and complication risk in genotype-driven and multifactorial settings. In order to serve as in vitro diagnostic or clinical decision-support tools, iPSC-ECs preparations would need to be qualified using predefined acceptance criteria for endothelial identity, purity, genomic integrity, and assay-specific functional performance, with reproducibility demonstrated across differentiations and appropriate reference controls.

Moreover, emerging research highlights that iPSC disease modeling offers a revolutionary approach to understanding how environmental toxins impact human health. Leveraging iPSCs, scientists can generate highly specific, organ-like tissues—such as cardiac muscle or complex organoids—that faithfully replicate native tissue architecture and function. This innovative method allows for the exploration of gene–environment interactions, particularly how genetic polymorphisms may increase individual susceptibility to environmental toxins. Through iPSC-based models, researchers can uncover critical insights into how these interactions exacerbate cardiovascular disease and contribute to adverse health outcomes. The integration of advanced techniques like single-cell RNA sequencing, mass spectrometry, and comprehensive -omic analyses provides detailed insights into how environmental factors disrupt cellular functions at a high resolution.

This proactive approach not only could facilitate early detection of toxic effects and pathogenic processes before they manifest in population-wide epidemiological patterns—supporting early intervention and prevention—but also accelerate the discovery of biomarkers and therapies, thereby advancing predictive and personalized medicine.

## Figures and Tables

**Table 1:** Summary of the applications of patient-specific iPSC-ECs as predictive and personalized models for cardiovascular diseases and associated conditions.

Cardiovascular Diseases and Associated Conditions	iPSC-ECs as Predictive Models	iPSC-ECs as Personalized Models
Familiar hypercholesterolemia (FH)	Patient-derived iPSC-ECs were found to display reduced expression of LDL receptors, impaired angiogenic capacity, and heightened inflammatory and oxidative stress signatures, regardless of cholesterol levels [34]	Expression levels of mature LDL receptors in iPSC-ECs depend on specific patient mutations [34]
Anti-complement factor H autoantibody-associated atypical hemolytic uremic syndrome (aHUS)	Patient-derived ECs exhibit intrinsic endothelial defects as complement-mediated cytotoxicity, increased apoptosis rates, and exacerbated inflammatory signaling, thus recreating the pathological phenotype. Activation of p38 signaling reconstitutes endothelial integrity [39]	-
Diabetic endothelial dysfunction	ECs derived from diabetic patient iPSCs expressed inflammation-related markers, higher intracellular ROS levels, and a higher proportion of platelet-adherent cells compared to controls. Interestingly, data show that unhealthy lines may better tolerate an environment which simulates diabetic plasma [46]	It is possible to develop patient-specific drug therapies to restore endothelial functionality [46]
Obesity	iPSC-ECs from diet-induced obesity mice exhibited significantly reduced migration and NO production, while increasing apoptosis and inflammation. Pravastatin significantly reduced the occurrence of these dysfunctions in both in vitro and in vivo models [47]	-
Pulmonary arterial hypertension	iPSC-ECs from different patients had reduced survival under stress, impaired angiogenesis, reduced adhesion to extracellular matrices, and decreased migration. AG1296 enhanced survival by restoring BMPR2 signaling, as also confirmed with in vivo analyses [48] (BMPR2 = Bone morphogenic receptor − 2)	-
Cadmium toxicity	By exposing H9-ECs to cadmium chloride, it is possible to observe reduced EC proliferation and angiogenic capacity. Inhibiting p38 and ERK pathways, EC function can be partially restored [51] (H9-ECs = H9 human embryonic stem cells − derived endothelial cells)	-
Coronary artery disease	Following endothelial differentiation, iPSCs from different coronaropathic patients all show increased epigenetic “mitotic age” estimates derived from DNA methylation [53]	ECs from young patients with acute coronary syndrome tend to show stronger TNF-α-induced upregulation of adhesion molecules and an increased monocyte recruitment [53], while ECs from old high-risk donors display a more rapid telomere shortening [54]

**Table 2:** Summary of the applications of patient-specific iPSC-ECs as predictive and personalized models for cerebrovascular diseases and associated conditions.

Cerebrovascular Diseases and Associated Conditions	iPSC-ECs as Predictive Models	iPSC-ECs as Personalized Models
Cerebral Autosomal Dominant Arteriopathy with Subcortical Infarcts and Leukoencephalopathy (CADASIL)	Co-cultured CADASIL–VSMCs and CADASIL–ECs show an inability to support microvessel network formation. Adding VEGF and knocking down mutant NOTCH3 partially restores capillary stability [56] (VSMC = vascular smooth muscle cell)	CADASIL cells can be tested with various Notch pathway modulators or with metabolic treatments to guide therapy tailored to every single patient’s molecular defect
Moyamoya disease (MMD)	MMD patient-derived iPSC-ECs form fewer, shorter tube-like structures than controls, even under pro-angiogenic conditions, but without impeding endothelial growth (mitosis-related genes were actually upregulated) [58]. Nevertheless, endothelial structure is compromised in MMD-ECs, while MMD-VSMCs have fairly no differences compared to controls [61]	-
Familial cerebral cavernous malformation	Patient-specific organoids develop abnormally dilated, leaky vascular structures resembling cavernomas, showing compromised endothelial barrier integrity. Interestingly, patient-derived ECs can successfully integrate into ex vivo mouse brain explants by reshaping and remodeling pre-existing vessels, leading to increased vessel diameter and reorganization of nearby vascular smooth muscle cells [62]	-

## Data Availability

No new data were created or analyzed in this study. Data sharing is not applicable to this article.

## Abbreviations

The following abbreviations are used in this manuscript:

aHUS	Atypical hemolytic uremic syndrome
BMP	Bone morphogenetic protein
CADASIL	Cerebral Autosomal Dominant Arteriopathy with Subcortical Infarcts and Leukoencephalopathy
ECs	Endothelial cells
eNOS	Endothelial nitric oxide synthase
FH	Familial hypercholesterolemia
H9-ECs	H9 human embryonic stem cell-derived endothelial cells
hiPSC-ECs	Human-induced pluripotent stem cell-derived endothelial cells
MMD	Moyamoya disease
MMP	Matrix metalloproteinases
PRC	Polycomb Repressive Complex
TEnCs	Tumor-associated endothelial cells
VSMCs	Vascular smooth muscle cells
vWF	Von Willebrand factor

## References

[1] Trimm E., Red-Horse K. (2023). Vascular Endothelial Cell Development and Diversity. Nat. Rev. Cardiol..

[2] Petrova T.V., Koh G.Y. (2020). Biological Functions of Lymphatic Vessels. Science.

[3] Krüger-Genge A., Blocki A., Franke R.-P., Jung F. (2019). Vascular Endothelial Cell Biology: An Update. Int. J. Mol. Sci..

[4] dela Paz N.G., D’Amore P.A. (2009). Arterial versus Venous Endothelial Cells. Cell Tissue Res..

[5] Wu D., Chen Q., Chen X., Han F., Chen Z., Wang Y. (2023). The Blood–Brain Barrier: Structure, Regulation and Drug Delivery. Signal Transduct. Target. Ther..

[6] Favero G., Paganelli C., Buffoli B., Rodella L.F., Rezzani R. (2014). Endothelium and Its Alterations in Cardiovascular Diseases: Life Style Intervention. BioMed Res. Int..

[7] Gimbrone M.A., García-Cardeña G. (2016). Endothelial Cell Dysfunction and the Pathobiology of Atherosclerosis. Circ. Res..

[8] Nguyen D.V., Shaw L.C., Grant M.B. (2012). Inflammation in the Pathogenesis of Microvascular Complications in Diabetes. Front. Endocrinol..

[9] Kurakula K., Smolders V.F.E.D., Tura-Ceide O., Jukema J.W., Quax P.H.A., Goumans M.-J. (2021). Endothelial Dysfunction in Pulmonary Hypertension: Cause or Consequence?. Biomedicines.

[10] Liu L., Zhao B., Yu Y., Gao W., Liu W., Chen L., Xia Z., Cao Q. (2024). Vascular Aging in Ischemic Stroke. J. Am. Heart Assoc..

[11] Mekala A., Qiu H. (2025). Interplay Between Vascular Dysfunction and Neurodegenerative Pathology: New Insights into Molecular Mechanisms and Management. Biomolecules.

[12] Moncada S., Higgs E.A. (2006). The Discovery of Nitric Oxide and Its Role in Vascular Biology. Br. J. Pharmacol..

[13] Cai H., Harrison D.G. (2000). Endothelial Dysfunction in Cardiovascular Diseases: The Role of Oxidant Stress. Circ. Res..

[14] Chistiakov D.A., Orekhov A.N., Bobryshev Y.V. (2015). Endothelial Barrier and Its Abnormalities in Cardiovascular Disease. Front. Physiol..

[15] Paik D.T., Chandy M., Wu J.C. (2020). Patient and Disease–Specific Induced Pluripotent Stem Cells for Discovery of Personalized Cardiovascular Drugs and Therapeutics. Pharmacol. Rev..

[16] Nashun B., Hill P.W., Hajkova P. (2015). Reprogramming of Cell Fate: Epigenetic Memory and the Erasure of Memories Past. EMBO J..

[17] Sriram G., Tan J.Y., Islam I., Rufaihah A.J., Cao T. (2015). Efficient Differentiation of Human Embryonic Stem Cells to Arterial and Venous Endothelial Cells under Feeder- and Serum-Free Conditions. Stem Cell Res. Ther..

[18] Qiu J., Hirschi K.K. (2019). Endothelial Cell Development and Its Application to Regenerative Medicine. Circ. Res..

[19] Chen D., Schwartz M.A., Simons M. (2021). Developmental Perspectives on Arterial Fate Specification. Front. Cell Dev. Biol..

[20] Aranguren X.L., Beerens M., Coppiello G., Wiese C., Vandersmissen I., Lo Nigro A., Verfaillie C.M., Gessler M., Luttun A. (2013). COUP-TFII Orchestrates Venous and Lymphatic Endothelial Identity by Homo- or Hetero-Dimerisation with PROX1. J. Cell Sci..

[21] Arora S., Yim E.K.F., Toh Y.-C. (2019). Environmental Specification of Pluripotent Stem Cell Derived Endothelial Cells Toward Arterial and Venous Subtypes. Front. Bioeng. Biotechnol..

[22] Selim M.S., Matani B.R., Henry-Ojo H.O., Narayanan S.P., Somanath P.R. (2025). Claudin 5 Across the Vascular Landscape: From Blood–Tissue Barrier Regulation to Disease Mechanisms. Cells.

[23] Goncharov N.V., Popova P.I., Avdonin P.P., Kudryavtsev I.V., Serebryakova M.K., Korf E.A., Avdonin P.V. (2020). Markers of Endothelial Cells in Normal and Pathological Conditions. Biochem. Suppl. Ser. A Membr. Cell Biol..

[24] de Boer S., Laan S., Dirven R., Eikenboom J. (2024). Approaches to Induce the Maturation Process of Human Induced Pluripotent Stem Cell Derived-Endothelial Cells to Generate a Robust Model. PLoS ONE.

[25] Belt H., Koponen J.K., Kekarainen T., Puttonen K.A., Mäkinen P.I., Niskanen H., Oja J., Wirth G., Koistinaho J., Kaikkonen M.U. (2018). Temporal Dynamics of Gene Expression During Endothelial Cell Differentiation from Human IPS Cells: A Comparison Study of Signalling Factors and Small Molecules. Front. Cardiovasc. Med..

[26] Lanner F., Sohl M., Farnebo F. (2007). Functional Arterial and Venous Fate Is Determined by Graded VEGF Signaling and Notch Status During Embryonic Stem Cell Differentiation. Arterioscler. Thromb. Vasc. Biol..

[27] Gerhold K.A., Schwartz M.A. (2016). Ion Channels in Endothelial Responses to Fluid Shear Stress. Physiology.

[28] Beverley K.M., Ahn S.J., Levitan I. (2025). Flow-sensitive ion channels in vascular endothelial cells: Mechanisms of activation and roles in mechanotransduction. Biophys. J..

[29] Abutaleb N.O., Truskey G.A. (2021). Differentiation and characterization of human iPSC-derived vascular endothelial cells under physiological shear stress. STAR Protoc..

[30] Carcamo-Orive I., Hoffman G.E., Cundiff P., Beckmann N.D., D’Souza S.L., Knowles J.W., Patel A., Hendry C., Papatsenko D., Abbasi F. (2017). Analysis of Transcriptional Variability in a Large Human IPSC Library Reveals Genetic and Non-Genetic Determinants of Heterogeneity. Cell Stem Cell.

[31] Kim K., Doi A., Wen B., Ng K., Zhao R., Cahan P., Kim J., Aryee M.J., Ji H., Ehrlich L.I.R. (2010). Epigenetic Memory in Induced Pluripotent Stem Cells. Nature.

[32] Manganelli M., Mazzoldi E.L., Ferraro R.M., Pinelli M., Parigi M., Aghel S.A.M., Bugatti M., Collo G., Stocco G., Vermi W. (2024). Progesterone receptor is constitutively expressed in induced Pluripotent Stem Cells (iPSCs). Stem Cell Rev. Rep..

[33] Jiménez-Torres J.A., Virumbrales-Muñoz M., Sung K.E., Lee M.H., Abel E.J., Beebe D.J. (2019). Patient-Specific Organotypic Blood Vessels as an in Vitro Model for Anti-Angiogenic Drug Response Testing in Renal Cell Carcinoma. EBioMedicine.

[34] Zakharova I.S., Shevchenko A.I., Arssan M.A., Sleptcov A.A., Nazarenko M.S., Zarubin A.A., Zheltysheva N.V., Shevchenko V.A., Tmoyan N.A., Saaya S.B. (2024). IPSC-Derived Endothelial Cells Reveal LDLR Dysfunction and Dysregulated Gene Expression Profiles in Familial Hypercholesterolemia. Int. J. Mol. Sci..

[35] Brown M.S., Goldstein J.L. (1974). Familial Hypercholesterolemia: Defective Binding of Lipoproteins to Cultured Fibroblasts Associated with Impaired Regulation of 3-Hydroxy-3-Methylglutaryl Coenzyme a Reductase Activity. Proc. Natl. Acad. Sci. USA.

[36] Nair P. (2013). Brown and Goldstein: The Cholesterol Chronicles. Proc. Natl. Acad. Sci. USA.

[37] Chrzanowska-Wodnicka M. (2017). Rap1 in Endothelial Biology. Curr. Opin. Hematol..

[38] Singh B., Kosuru R., Lakshmikanthan S., Sorci-Thomas M.G., Zhang D.X., Sparapani R., Vasquez-Vivar J., Chrzanowska M. (2021). Endothelial Rap1 (Ras-Association Proximate 1) Restricts Inflammatory Signaling to Protect from the Progression of Atherosclerosis. Arterioscler. Thromb. Vasc. Biol..

[39] Zhou D., Tan Y., Liu X., Tang L., Wang H., Shen J., Wang W., Zhuang L., Tao J., Su J. (2021). Patient-Specific IPSC-Derived Endothelial Cells Reveal Aberrant P38 MAPK Signaling in Atypical Hemolytic Uremic Syndrome. Stem Cell Rep..

[40] Kavanagh D., Goodship T.H.J. (2011). Atypical Hemolytic Uremic Syndrome, Genetic Basis, and Clinical Manifestations. Hematology.

[41] Chu L.-Y., Wang Y.-F., Cheng H.-H., Kuo C.-C., Wu K.K. (2016). Endothelium-Derived 5-Methoxytryptophan Protects Endothelial Barrier Function by Blocking P38 MAPK Activation. PLoS ONE.

[42] Dejana E., Orsenigo F., Lampugnani M.G. (2008). The Role of Adherens Junctions and VE-Cadherin in the Control of Vascular Permeability. J. Cell Sci..

[43] Cao S., Reece E.A., Shen W.-B., Yang P. (2020). Restoring BMP4 Expression in Vascular Endothelial Progenitors Ameliorates Maternal Diabetes-Induced Apoptosis and Neural Tube Defects. Cell Death Dis..

[44] Mace K.A., Hansen S.L., Myers C., Young D.M., Boudreau N. (2005). HOXA3 Induces Cell Migration in Endothelial and Epithelial Cells Promoting Angiogenesis and Wound Repair. J. Cell Sci..

[45] Sauteur L., Krudewig A., Herwig L., Ehrenfeuchter N., Lenard A., Affolter M., Belting H.-G. (2014). Cdh5/VE-Cadherin Promotes Endothelial Cell Interface Elongation via Cortical Actin Polymerization during Angiogenic Sprouting. Cell Rep..

[46] Gorashi R., Rivera-Bolanos N., Dang C., Chai C., Kovacs B., Alharbi S., Ahmed S.S., Goyal Y., Ameer G., Jiang B. (2023). Modeling Diabetic Endothelial Dysfunction with Patient-specific Induced Pluripotent Stem Cells. Bioeng. Transl. Med..

[47] Gu M., Mordwinkin N.M., Kooreman N.G., Lee J., Wu H., Hu S., Churko J.M., Diecke S., Burridge P.W., He C. (2015). Pravastatin Reverses Obesity-Induced Dysfunction of Induced Pluripotent Stem Cell-Derived Endothelial Cells via a Nitric Oxide-Dependent Mechanism. Eur. Heart J..

[48] Gu M., Donato M., Guo M., Wary N., Miao Y., Mao S., Saito T., Otsuki S., Wang L., Harper R.L. (2021). IPSC–Endothelial Cell Phenotypic Drug Screening and in Silico Analyses Identify Tyrphostin-AG1296 for Pulmonary Arterial Hypertension. Sci. Transl. Med..

[49] Chandy M., Obal D., Wu J.C. (2022). Elucidating Effects of Environmental Exposure Using Human-induced Pluripotent Stem Cell Disease Modeling. EMBO Mol. Med..

[50] Sgromo C., Cucci A., Venturin G., Follenzi A., Olgasi C. (2024). Bridging the Gap: Endothelial Dysfunction and the Role of IPSC-Derived Endothelial Cells in Disease Modeling. Int. J. Mol. Sci..

[51] Tang L., Su J., Liang P. (2017). Modeling Cadmium-Induced Endothelial Toxicity Using Human Pluripotent Stem Cell-Derived Endothelial Cells. Sci. Rep..

[52] Tellez-Plaza M., Navas-Acien A., Menke A., Crainiceanu C.M., Pastor-Barriuso R., Guallar E. (2012). Cadmium Exposure and All-Cause and Cardiovascular Mortality in the U.S. General Population. Environ. Health Perspect..

[53] Straessler E.T., Kraenkel N., Landmesser U. (2022). RNA-Sequencing Reveals Significant Differences in the Inflammatory Response of IPSC-Derived Endothelial Cells from ACS Patients and Healthy Controls. Eur. Heart J..

[54] Straessler E.T., Kiamehr M., Aalto-Setala K., Kraenkel N.K., Landmesser U.L. (2020). IPSC-Derived Endothelial Cells Reflect Accelerated Senescence and Increased Inflammatory Response in a CVD-Risk Stratified Manner. Eur. Heart J..

[55] Chabriat H., Joutel A., Dichgans M., Tournier-Lasserve E., Bousser M.-G. (2009). CADASIL. Lancet Neurol..

[56] Zhang W., Zhao X., Qi X., Kimber S.J., Hooper N.M., Wang T. (2023). Induced Pluripotent Stem Cell Model Revealed Impaired Neurovascular Interaction in Genetic Small Vessel Disease Cerebral Autosomal Dominant Arteriopathy with Subcortical Infarcts and Leukoencephalopathy. Front. Cell Neurosci..

[57] Ihara M., Yamamoto Y., Hattori Y., Liu W., Kobayashi H., Ishiyama H., Yoshimoto T., Miyawaki S., Clausen T., Bang O.Y. (2022). Moyamoya Disease: Diagnosis and Interventions. Lancet Neurol..

[58] Hitomi T., Habu T., Kobayashi H., Okuda H., Harada K.H., Osafune K., Taura D., Sone M., Asaka I., Ameku T. (2013). Downregulation of Securin by the Variant RNF213 R4810K (Rs112735431, G & gt; A) Reduces Angiogenic Activity of Induced Pluripotent Stem Cell-Derived Vascular Endothelial Cells from Moyamoya Patients. Biochem. Biophys. Res. Commun..

[59] Hamauchi S., Shichinohe H., Uchino H., Yamaguchi S., Nakayama N., Kazumata K., Osanai T., Abumiya T., Houkin K., Era T. (2016). Cellular Functions and Gene and Protein Expression Profiles in Endothelial Cells Derived from Moyamoya Disease-Specific IPS Cells. PLoS ONE.

[60] Stupack D.G., Cheresh D.A. (2004). Integrins and Angiogenesis. Curr. Top. Dev. Biol..

[61] Tokairin K., Hamauchi S., Ito M., Kazumata K., Sugiyama T., Nakayama N., Kawabori M., Osanai T., Houkin K. (2020). Vascular Smooth Muscle Cell Derived from IPS Cell of Moyamoya Disease-Comparative Characterization with Endothelial Cell Transcriptome. J. Stroke Cerebrovasc. Dis..

[62] Arce M., Erzar I., Yang F., Senthilkumar N., Onyeogaziri F.C., Ronchi D., Ahlstrand F.C., Noll N., Lugano R., Richards M. (2025). KRIT1 Heterozygous Mutations Are Sufficient to Induce a Pathological Phenotype in Patient-Derived IPSC Models of Cerebral Cavernous Malformation. Cell Rep..

[63] Queisser A., Seront E., Boon L.M., Vikkula M. (2021). Genetic Basis and Therapies for Vascular Anomalies. Circ. Res..

[64] Bassett A.R. (2017). Editing the genome of hiPSC with CRISPR/Cas9: Disease models. Mamm. Genome.

[65] Cerneckis J., Cai H., Shi Y. (2024). Induced Pluripotent Stem Cells (IPSCs): Molecular Mechanisms of Induction and Applications. Signal Transduct. Target. Ther..

[66] Li G., Gao J., Ding P., Gao Y. (2025). The Role of Endothelial Cell–Pericyte Interactions in Vascularization and Diseases. J. Adv. Res..

[67] Méndez-Barbero N., Gutiérrez-Muñoz C., Blanco-Colio L. (2021). Cellular Crosstalk between Endothelial and Smooth Muscle Cells in Vascular Wall Remodeling. Int. J. Mol. Sci..

[68] Pober J.S., Sessa W.C. (2007). Evolving Functions of Endothelial Cells in Inflammation. Nat. Rev. Immunol..

[69] Schaeffer S., Iadecola C. (2021). Revisiting the Neurovascular Unit. Nat. Neurosci..

[70] Segarra M., Aburto M.R., Hefendehl J., Acker-Palmer A. (2019). Neurovascular Interactions in the Nervous System. Annu. Rev. Cell Dev. Biol..

[71] Vozzi F., Campolo J., Cozzi L., Politano G., Di Carlo S., Rial M., Domenici C., Parodi O. (2018). Computing of Low Shear Stress-Driven Endothelial Gene Network Involved in Early Stages of Atherosclerotic Process. BioMed Res. Int..

[72] Ceccherini E., Persiani E., Cabiati M., Guiducci L., Del Ry S., Gisone I., Falleni A., Cecchettini A., Vozzi F. (2024). A Dynamic Cellular Model as an Emerging Platform to Reproduce the Complexity of Human Vascular Calcification In Vitro. Int. J. Mol. Sci..

[73] Gisone I., Boffito M., Persiani E., Pappalardo R., Ceccherini E., Alliaud A., Cabiati M., Laurano R., Guiducci L., Caselli C. (2025). Integration of Co-Culture Conditions and 3D Gelatin Methacryloyl Hydrogels to Improve Human-Induced Pluripotent Stem Cells-Derived Cardiomyocytes Maturation. Front. Bioeng. Biotechnol..

[74] Aoki H., Yamashita M., Hashita T., Ogami K., Hoshino S., Iwao T., Matsunaga T. (2020). Efficient differentiation and purification of human induced pluripotent stem cell-derived endothelial progenitor cells and expansion with the use of inhibitors of ROCK, TGF-β, and GSK3β. Heliyon.

[75] Rabino M., Sommariva E., Zacchigna S., Pompilio G. (2023). From bedside to the bench: Patient-specific hiPSC-EC models uncover endothelial dysfunction in genetic cardiomyopathies. Front. Physiol..

[76] Helle E., Ampuja M., Dainis A., Antola L., Temmes E., Tolvanen E., Mervaala E., Kivelä R. (2021). HiPS-Endothelial Cells Acquire Cardiac Endothelial Phenotype in Co-culture with hiPS-Cardiomyocytes. Front. Cell Dev. Biol..

[77] Wang Y.T., Meng X.T. (2023). A review of the evidence to support electrical stimulation-induced vascularization in engineered tissue. Regen. Ther..

[78] Shakeri A., Wang Y., Zhao Y., Landau S., Perera K., Lee J., Radisic M. (2023). Engineering Organ-on-a-Chip Systems for Vascular Diseases. Arterioscler. Thromb. Vasc. Biol..

[79] Nair A.L., Groenendijk L., Overdevest R., Fowke T.M., Annida R., Mocellin O., de Vries H.E., Wevers N.R. (2023). Human BBB-on-a-chip reveals barrier disruption, endothelial inflammation, and T cell migration under neuroinflammatory conditions. Front. Mol. Neurosci..

[80] Fontanelli L., Nisini N., Pirola S., Recchia F.A. (2025). Neuromuscular and Cardiac Organoids and Assembloids: Advanced Platforms for Drug Testing. Pharmacol. Ther..

